# Leukemia Gene Atlas – A Public Platform for Integrative Exploration of Genome-Wide Molecular Data

**DOI:** 10.1371/journal.pone.0039148

**Published:** 2012-06-14

**Authors:** Katja Hebestreit, Sören Gröttrup, Daniel Emden, Jannis Veerkamp, Christian Ruckert, Hans-Ulrich Klein, Carsten Müller-Tidow, Martin Dugas

**Affiliations:** 1 Institute of Medical Informatics, University of Muenster, Muenster. Germany; 2 Department of Medicine, Hematology and Oncology, University of Muenster, Muenster, Germany; University of Thessaly, Faculty of Medicine, Greece

## Abstract

Leukemias are exceptionally well studied at the molecular level and a wealth of high-throughput data has been published. But further utilization of these data by researchers is severely hampered by the lack of accessible integrative tools for viewing and analysis. We developed the Leukemia Gene Atlas (LGA) as a public platform designed to support research and analysis of diverse genomic data published in the field of leukemia. With respect to leukemia research, the LGA is a unique resource with comprehensive search and browse functions. It provides extensive analysis and visualization tools for various types of molecular data. Currently, its database contains data from more than 5,800 leukemia and hematopoiesis samples generated by microarray gene expression, DNA methylation, SNP and next generation sequencing analyses. The LGA allows easy retrieval of large published data sets and thus helps to avoid redundant investigations. It is accessible at www.leukemia-gene-atlas.org.

## Introduction

Recent advances in high-throughput technologies allow to collect unprecedented amounts of genomic, trancriptomic and epigenomic data. Even single studies can be based on genome wide microarray expression data of more than 2 000 patients [1]. Novel sources of high-throughput data such as those based on next generation sequencing promise to further enhance molecular analyses of leukemias on a genome wide level [2], [3]. High-throughput data are usually submitted to a public repository where they can be accessed and used for further analyses. These data have the potential to substantially accelerate and enhance further research [4], [5]. For example, for newly identified inactivating mutations or gene deletions it is of interest to identify gene expression patterns across hematopoietic differentiation and in different hematological malignancies. Furthermore, comparison of a new data set with published data can confirm results and accelerate discoveries [6]. Rapid and reliable access to published data sets can therefore save costs and speed up research. However, the access to published data by non-bioinformaticians is time-consuming, error-prone and often outright not successful. Thus, there is a need for a repository that enables researchers to retrieve information from already published data and helps to avoid redundant investigations [7]. The requirements for such a repository include the following: It should contain a wide range of molecular data types. The samples corresponding to the data should be annotated thoroughly with regard to leukemia, both clinically and biologically. The repository should provide search and browse functions as well as analysis and visualization tools to process the data. Besides, the repository should be freely accessible.

Here, we describe the Leukemia Gene Atlas (LGA), a novel online bioinformatics tool that provides comprehensive, easy and fast access to published genome wide data sets in hematopoiesis and hematological malignancies. In the following section we describe the architecture of the LGA paying particular attention to the database and the data stored therein. The primary purpose of the LGA is to support translational research and biomarker discovery in hematology.

## Materials and Methods

**Figure 1:**
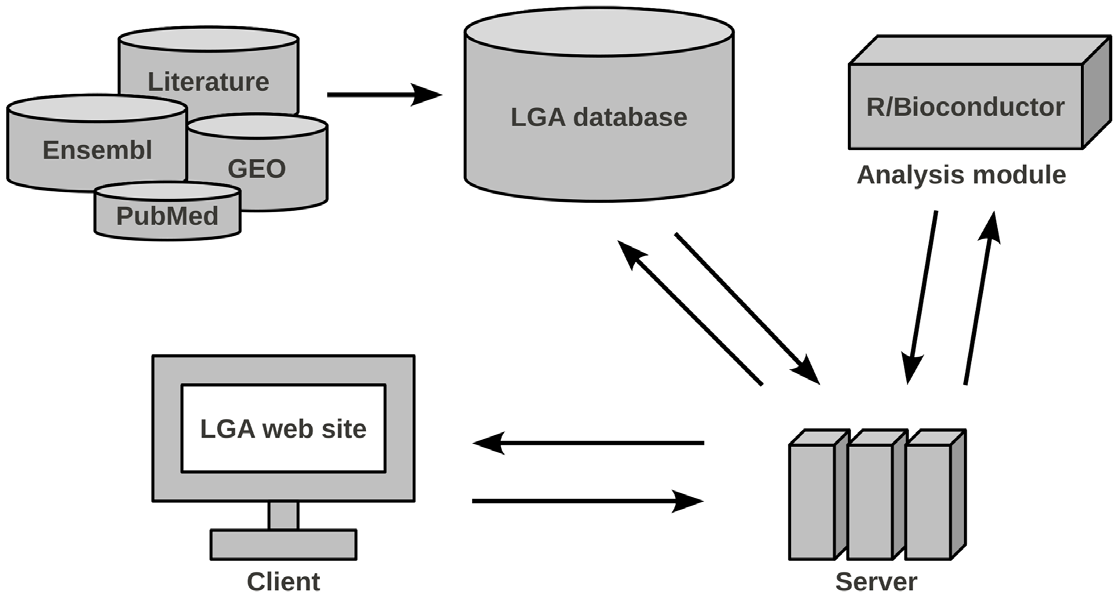
Overview of the LGA architecture. Data is imported from several online repositories and the medical literature into the LGA database. An analysis module processes the molecular data. The application server handles data transfer between database and analysis module and can be accessed through a web interface. It executes queries and forwards data and analysis results to the client.

The LGA consists of three components: database, data analysis module and web-based user-interface, Figure 1. The database stores the molecular data together with all available information from publications and constitutes the centerpiece of the LGA. This database can be accessed using search functions by a user-friendly web front-end. This front-end also allows conducting data analyses. In the following sections these components are described in more detail.

### The Database

The database (PostgreSQL [8]) scheme is kept flexible to include biologically and technically highly diverse experiments, Table 1. Currently, the database contains studies based on DNA-methylation, gene expression, copy number/genotype, and next-generation sequencing data. These studies focus on different aspects such as prediction of molecular subtypes of leukemias, research of human hematopoiesis and the analysis of transcription factor binding sites. The majority of these molecular data was imported from Gene Expression Omnibus (GEO) [9] and new data sets are continuously added. Data published in peer-reviewed journals only is considered to be integrated. And only after passing a quality control and, if necessary, additional preprocessing steps, the molecular data is added semi-automatically. Data preprocessing and import into the database are generally done in R/Bioconductor [10], [11]. In addition to the molecular data, basic information about the underlying experiments is stored as well as a link to the related publications. Clinical and biological characteristics of the respective samples, patients and cell lines are deposited as well. Considerable effort was made to extract as many attributes as possible, particularly with regard to leukemias. For this purpose the sample characteristics arising from GEO were completed by further attributes obtained manually from the corresponding publication. Where available, survival data was also included. Currently, there are more than 30 clinical and biological attributes to describe samples and patients respectively.

**Table 1:** Overview of data in the LGA.

Publication	Samples	Experiment type	Sample size
Kohlmann et al. Leukemia 2010	AML	Gene expression (microarray)	251
Haferlach et al. J Clin Oncol 2010	ALL/AML/CLL/CML/MDS/healthy	Gene expression (microarray)	3248
Figueroa et al. Cancer cell 2010	AML/Healthy	DNA-methylation (microarray)	352
Verhaak et al. Haematologica 2010	AML	Gene expression (microarray)	461
Valk et al. N Engl J Med 2010	AML/Healthy	Gene expression (microarray)	293
Bullinger et al. Leukemia 2010	AML/Diagnosis/Remission	Genotype (microarray)	328
Kohlmann et al. J Clin Oncol 2010	CMML	DNA sequencing	81
Gutierrez et al. Leukemia 2005	AML	Gene expression (microarray)	43
Novershtern et al. Cell 2011	Human hematopoietic cells	Gene expression (microarray)	211
Figueroa et al. Cancer Cell 2010	AML/Healthy	Gene expression, DNA-methylation (microarray)	411
Kohlmann et al. Leukemia 2011	CMML	DNA sequencing	18
Tijssen et al. Developmental cell 2011	Primary human megakaryocytes	ChIP-sequencing	5
Eppert et al. Nat Med 2011	AML/Primary human cord blood	Gene expression (microarray)	105
Schenk et al. Nat Med 2012	Treated cell-lines (TEX/HL60)	Gene expression (microarray), ChIP-sequencing	30
Bruns et al. Leukemia 2009	Hematopoietic stem cells in CML	Gene expression (microarray)	47
Diaz-Blanco et al. Leukemia 2007	Hematopoietic stem cells in CML	Gene expression (microarray)	17

Apart from molecular data and its annotations, the database also includes important results arising from analyses of this molecular data. Results might be, for example, tables of differentially expressed genes, gene ontology terms or copy number alterations. Regarding next-generation sequencing studies, tables of discovered mutations or binding sites are deposited. These results are usually extracted from the articles and supplementary tables, or are generated by ourselves according to the data analysis description in the publication.

In addition, the result tables comprise an extract of the COSMIC database [12]. For each hematopoietic disease and investigated gene the number of samples which have been tested for mutations and the number of detected mutations in this gene are included.

### The Web Site

The LGA database is freely accessible via a web site (www.leukemia-gene-atlas.org) which supports selection and analysis of samples with comprehensive search and analysis functions. Data, result tables and generated graphics can be exported for further downstream analysis.

For each experiment, basic publication and data source information is provided as well as experimental details such as data type (e.g. gene expression or DNA methylation), platform used (e.g. which microarray or sequencer), and the number of analyzed samples.

Experiments can be filtered by sample or study characteristics, e.g. data type, leukemia subtype or karyotype. Via filters the user may create collections of samples by their biological and clinical characteristics. The data of defined collections can be analyzed and downloaded.

For some analysis functions it can be useful or necessary to specify genes of interest. User-defined lists of relevant genes or features (e.g. Affymetrix probe sets) can be added to the predefined ones, for instance genes associated with apoptosis or cell cycle.

Searching for genes and genome coordinates within result tables is a key functionality of the LGA. For example, groups of samples can be identified whose expression or methylation patterns significantly differ for certain genes of interest. In addition, the result search automatically scans a summary of the COSMIC database and displays the number of patients harboring mutations in the respective genes according to their hematopoietic disease. A hyperlink forwards the user to COSMIC Biomart [13] with filters set to the corresponding gene and disease.

### Data Analysis Tools

The web site provides a wide range of analysis tools for processing stored data.

To get insight into the distribution of measurement values across samples and groups of samples, bar charts are available with an integrated phenotype color grid as well as box plots. The phenotype color grid is an extension for visualization tools representing clinical and biological characteristics of the samples and enabling identification of possible correlations between phenotypes and molecular data.

Unsupervised analyses by means of principal component analysis and hierarchical clustering are available for exploration of gene expression and DNA-methylation data. Results of hierarchical clustering are presented by dendrograms together with a heat map where columns correspond to the samples and rows to the features of the platform. It is extended by the phenotype color grid to support the identification of potential subgroups of samples by their molecular data.

Testing for differential expression or DNA-methylation in groups of samples is possible via an ANOVA or Welch's t-test with adjustment for multiple testing [14].

Survival analysis is provided for data sets with available survival annotation. Samples can be grouped either by their molecular data (expression/DNA-methylation profile of a specific gene) or by their clinical and biological characteristics. Survival times of these groups of samples can be compared by Kaplan-Meier-Plots and log-rank test.

All data analysis functions are implemented in R/Bioconductor [10], [11].

As an established visualization tool we embedded the Integrative Genomics Viewer (IGV) [15]. It supports all data types of the LGA and enables interactive exploration of large data sets from multiple studies in parallel.

## Results

In the following, we demonstrate the usability of the LGA to generate or substantiate new hypotheses based on published genomic data sets. The presented example integrates ChIP-seq and gene expression data sets from four different studies. All methods and data are provided by the LGA and results were directly generated from the LGA web site.

**Figure 2:**
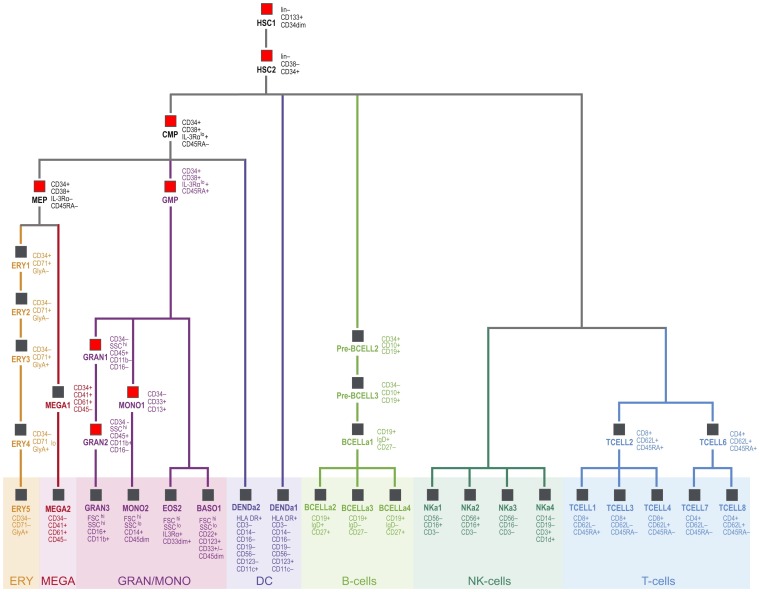
Populations of human hematopoietic cells. 38 hematopoietic cell populations are shown with their respective positions in hematopoiesis. Cells called as “progenitors” in the analysis are marked by a red box, “non-progenitor” cells are marked by a gray box. Figure adapted from Novershtern et al. [9].

**Figure 3:**
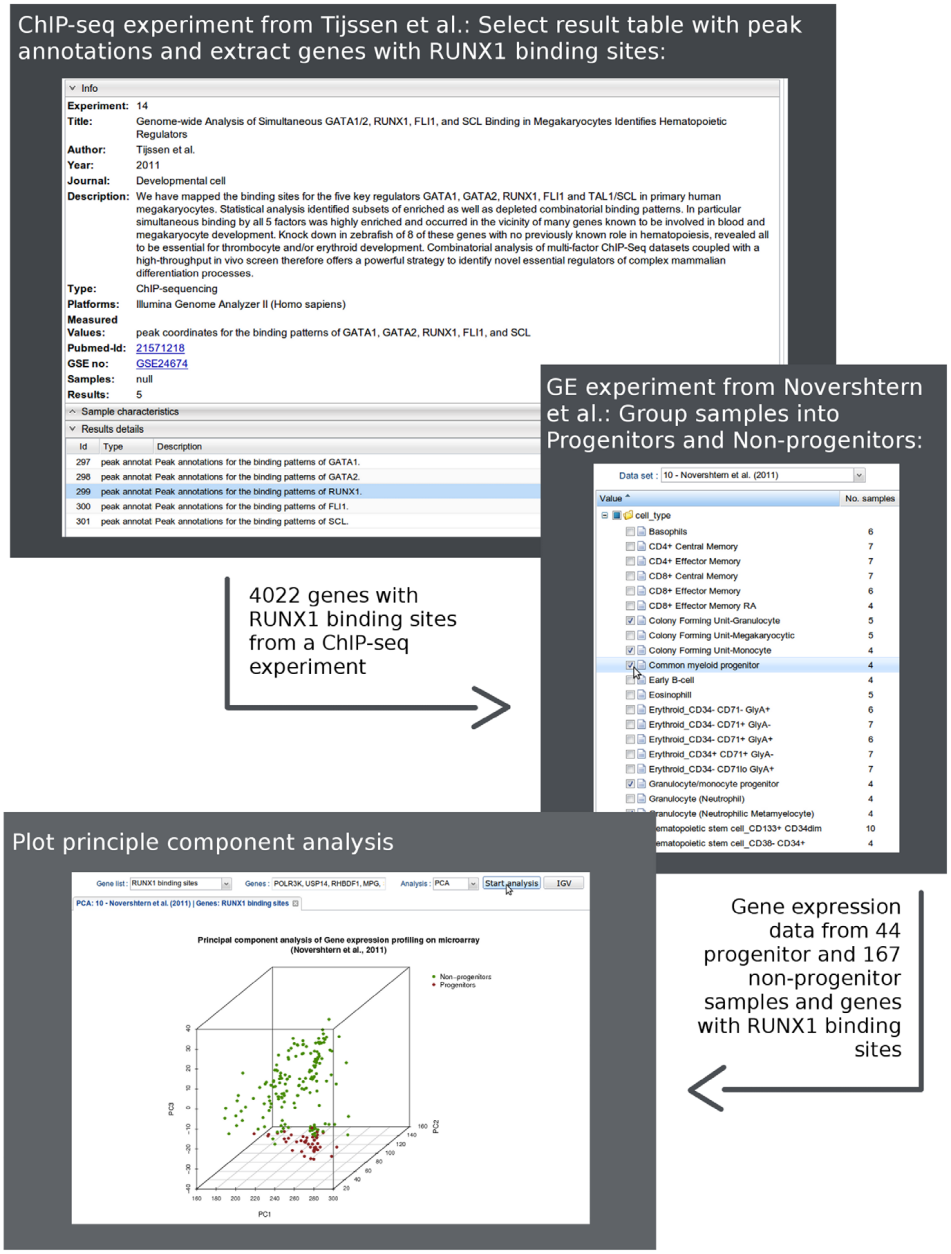
Usage of the LGA web interface. (Above) Experiment view with information on the integrated study [15] (above), sample characteristics (hidden, in the middle) and stored result tables (below). Genes with *RUNX1* binding sites are copied from a table of peak annotations and stored as a gene list. **(Middle)** Groups of samples from [14] are defined in the analysis tab. **(Below)** Selecting the stored gene list (genes with *RUNX1* binding sites) and performing principle component analysis on the selected groups of samples from [14].

**Figure 4:**
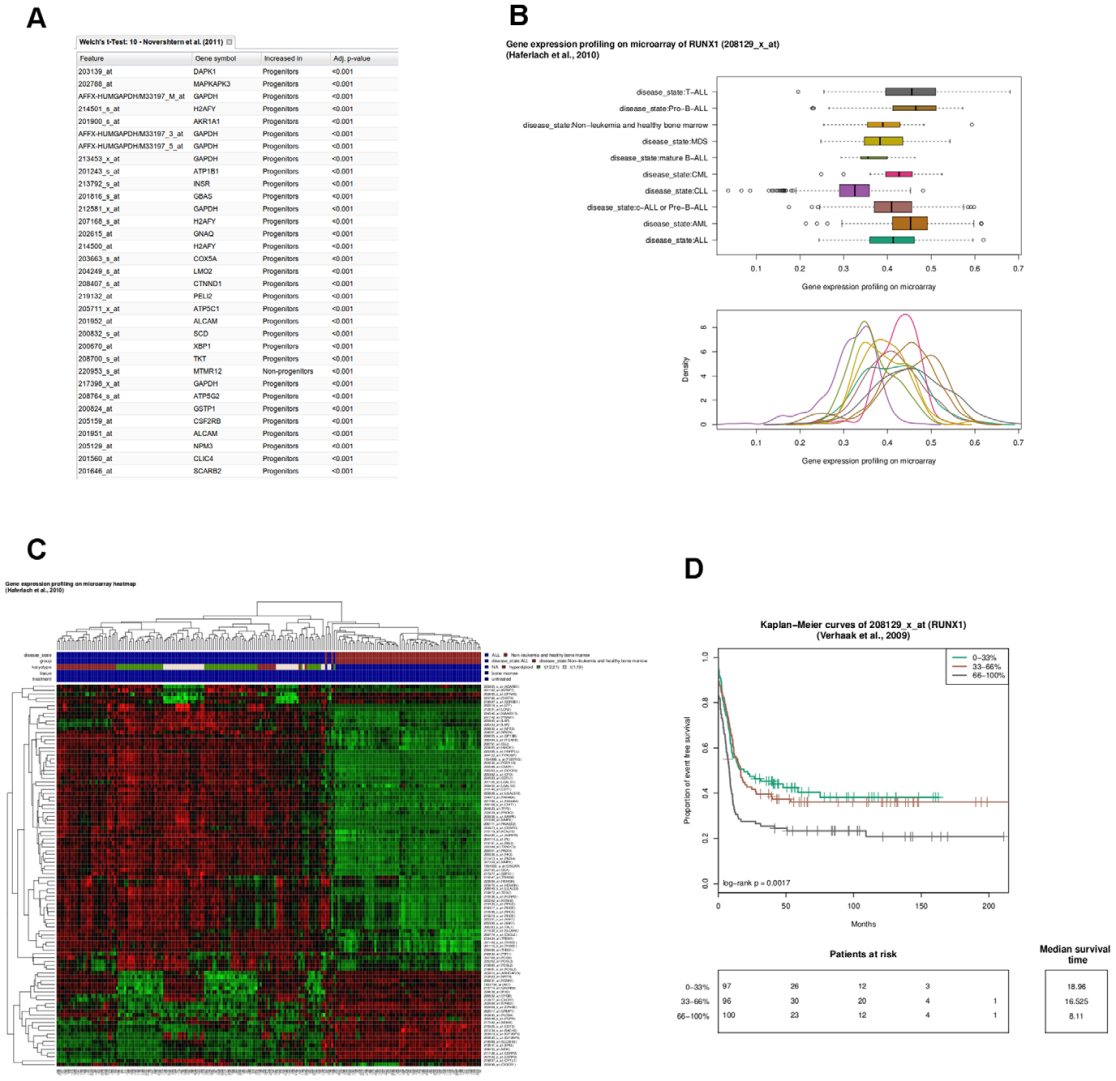
The role of *RUNX1* and its binding sites in leukemias. (**A**) Screenshot of a t-test result table with the 33 most differentially expressed genes with *RUNX1* binding sites in progenitor and non-progenitor cells. (**B**) Distribution of *RUNX1* expression for different leukemic disease states. (**C**) Heat map and hierarchical clustering of patients with acute lymphoblastic leukemia and non-leukemia samples with healthy bone marrows for gene expression of genes with RUNX1 binding sites and highest variances over all samples. The phenotype color grid at the top represents the sample characteristics. (**D**) Kaplan Meier curves of event-free survival for patients with acute myeloid leukemia with low (≤33% quantile), median (>33% quantile and ≤66% quantile), and high *RUNX1* expression (>66% quantile).

*RUNX1* is a regulatory gene in hematopoiesis and plays a key role in the development of leukemias [16]. To investigate the role of *RUNX1* in hematopoiesis we classified 38 distinct populations of human hematopoietic cells [17] into progenitors and non-progenitors (Figure 2). Next, we selected all genes that have a *RUNX1* binding site according to the ChIP-seq data set from Tijssen et al. [18]. Clustering based on the expression values of these *RUNX1* regulated genes separated the progenitor from the non-progenitor cells (Figure 3). T-tests revealed that 31 of the 33 most differentially expressed genes with *RUNX1* binding sites (FDR <0.001) were overexpressed in progenitors (Figure 4A). To investigate the role of *RUNX1* in leukemias we compared *RUNX1* expression for nine different leukemias and healthy controls in more than 2000 leukemia and control specimens derived from the MILE study [1]. *RUNX1* was notably down regulated in chronic lymphoid leukemia samples (Figure 4B). Hierarchical clustering based on all genes with *RUNX1* binding sites showed a strong subdivision of the samples into disease states, e.g. acute lymphoblastic leukemia separated from controls (Figure 4C; with the phenotype color grid).

Searching for *RUNX1* in published results across all studies revealed differential expression for groups of leukemias (Figure S1) and that mutations in *RUNX1* occur frequently. The extract of COSMIC shows that there are 90 *RUNX1* mutations in 688 patients with acute myeloid leukemia (Figure S2). In a sequencing study [19] seven different *RUNX1* mutations in chronic myelomonocytic leukemia samples have been detected. Six of these seven mutations are single nucleotide changes (Figure S2). A survival analysis of 293 patients with acute myeloid leukemia taken from Verhaak et al. [20] revealed an association between event-free survival and RUNX1 expression: a reduced expression of RUNX1 was associated with better outcome (Figure 4D).

## Discussion

In the literature, leukemia samples are thoroughly characterized in terms of mutation status and cytogenetics. Most repositories and databases lack the ability to make use of these important and helpful data. Gene Expression Omnibus (GEO) [9] has its limitations regarding queries and analyses. Queries for studies are currently possible via keywords only, specific leukemia related annotations are missing and analysis tools are not recommended for robust systematic analyses [9], [21]. Analyses provided in ArrayExpress [22] are currently limited to gene expression data and do not include the sample's karyotypes or mutations as condition query. User-defined custom analyses are currently not possible. Oncomine [23] is a commercial cancer microarray database storing results of differential expression analyses. Available gene signatures are predominantly restricted to the comparison of cancer vs. normal samples or a cancer subtype vs. all other subtypes and the user cannot perform analyses on alternative groups of samples. Other repositories, such as dbGAP database of genotypes and phenotypes [24], The Cancer Genome Atlas [25] and the Atlas of Genetics and Cytogenetics in Oncology and Heamatology [26] are less suitable for re-analysis and integration of published high-throughput data.

To our knowledge, the LGA is the first repository custom-tailored to the requirements of the leukemia research community in the field of molecular and clinical data. It provides extensive access to published leukemia data and thus helps to interpret newly measured data. It comprises several types of molecular data and supports integration of data types. The corresponding samples are annotated extensively. The user can choose between eight different analysis and visualization tools. Further data sets and data types, e.g. based on ChIP-chip or reduced representation bisulfite sequencing experiments, are continuously added.

Taken together, the LGA fills an urgent need for a usable and multifaceted repository for leukemia and hematopoiesis data sets. Its easy accessibility can enhance further leukemia research and biomarker development.

## Supporting Information

Figure S1 **Different** ***RUNX1*** **expression.** Screenshot of an extract of results for *RUNX1* search showing the groups of samples where *RUNX1* is differentially expressed for three experiments.
(TIF)

Figure S2 **Mutations in** ***RUNX1*** **.** Screenshot of an extract of results for *RUNX1* search showing detected mutations in patients with chronic myelomonocytic leukemia (above) and the number of detected mutations per disease state in COSMIC (below).
(TIF)
