# Supplementary figures and images for: Leukemia Gene Atlas – A Public Platform for Integrative Exploration of Genome-Wide Molecular Data
Source: PLoS One. 2012 Jun 14;7(6):e39148. doi: 10.1371/journal.pone.0039148 (PMC3375295; doi:10.1371/journal.pone.0039148)

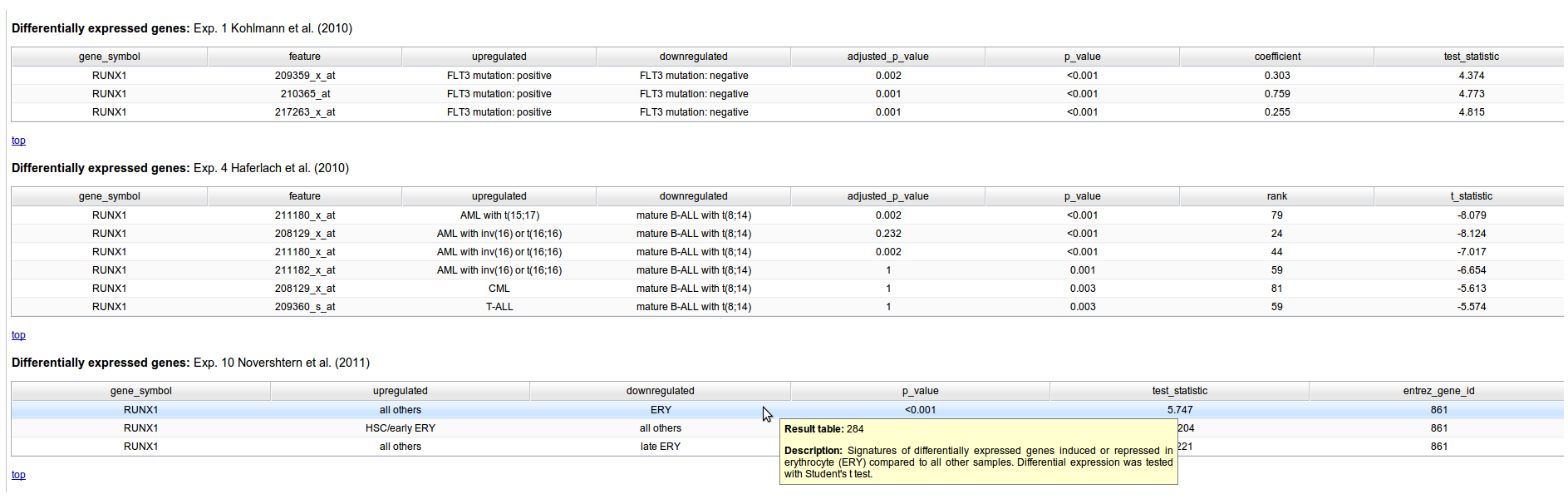

Supplement: Figure S1 — Different RUNX1 expression. Screenshot of an extract of results for RUNX1 search showing the groups of samples where RUNX1 is differentially expressed for three experiments. (TIF) [file pone.0039148.s001.tif]

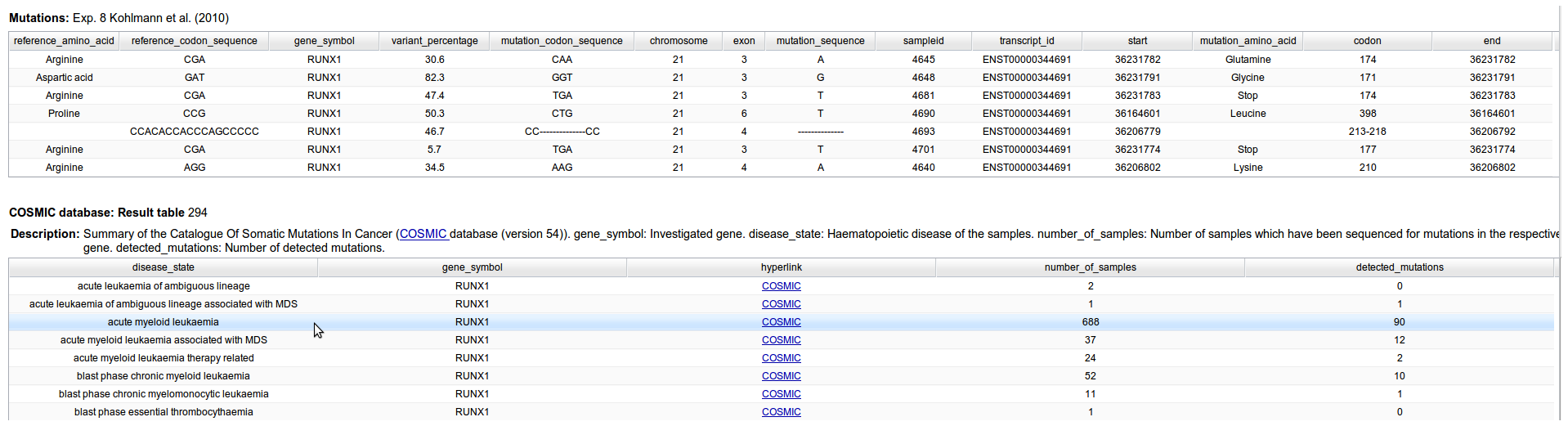

Supplement: Figure S2 — Mutations in RUNX1 . Screenshot of an extract of results for RUNX1 search showing detected mutations in patients with chronic myelomonocytic leukemia (above) and the number of detected mutations per disease state in COSMIC (below). (TIF) [file pone.0039148.s002.tif]
